# Feeding or starvation: which condition makes the red palm weevil, *Rhynchophorus ferrugineus* Olivier, more susceptible to insecticides?

**DOI:** 10.3389/finsc.2025.1743217

**Published:** 2026-01-16

**Authors:** Nagdy F. Abdel-Baky, Saleh S. Alhewairini, Saleem A. Alsalhee, Turki S. M. Alanazi, Raid R. Alharbi

**Affiliations:** 1 Department of Plant Protection, College of Agriculture and Food, Qassim University, Buraidah, Saudi Arabia; 2 Biological Control Agents and Bumblebee Production Center, The National Center for the Prevention and Control of Plant Pests and Animal Diseases “WEQAA“, Unaizah, Saudi Arabia; 3 Department of Plant Health, The National Center for the Prevention and Control of Plant Pests and Animal Diseases “WEQAA”, Riyadh, Saudi Arabia; 4 Saudi Authority for Industrial Cities and Technology Zones (MODON), Riyadh, Saudi Arabia

**Keywords:** sustainable pest management, red palm weevil, *Rhynchophorus ferrugineus*, chemical control strategies, insecticide efficacy, adult and larval susceptibility, feeding status, IPM

## Abstract

**Introduction:**

Over the last four decades, the red palm weevil (RPW), *Rhynchophorus ferrugineus*, has emerged as one of the most destructive pests of date and ornamental palms worldwide, causing major economic losses, with insecticide susceptibility strongly influenced by nutritional status.

**Methods:**

This study assessed how feeding versus starvation affects the sensitivity of RPW larvae and adults to ten commonly used insecticides at three dose levels (½×, 1×, and 2× of the recommended rate). Mortality rates were recorded and compared between fed and unfed insects.

**Results:**

Starvation significantly increased mortality, particularly in larvae, which were consistently more vulnerable than adults. Voliam Flexi achieved complete mortality under both conditions, whereas Coragen, Cyprone, and Indocarb caused full larval mortality only under starvation at the highest dose. In adults, 100% mortality with Medprid, Sivanto, Fiprol, and Deciban occurred only in starved insects. Feeding reduced insecticide toxicity, likely via enhanced detoxification or dilution of toxins through ingested sap, as indicated by lower LC₅₀ values in unfed insects for Sivanto, Coragen, Fedothrin, and Lamdoc.

**Discussion/Conclusion:**

These findings highlight feeding status as a critical determinant of insecticide efficacy. Pre-treatment starvation or natural food scarcity can enhance chemical performance, reduce insecticide use, and improve integrated pest management (IPM) strategies. The study also emphasizes the need for further research to elucidate the physiological mechanisms linking nutrition, detoxification, and insecticide susceptibility in RPW.” and confirmed as accurate.

## 1 Introduction

The red palm weevil (RPW), {*Rhynchophorus ferrugineus* Olivier; Coleoptera: Curculionidae} stands among the most destructive and economically devastating invasive pests of palms worldwide. RPW infests more than forty palm species, including both date and ornamental palms, and continues to cause immense agricultural and ecological losses across its expanding range (1–4). Beyond its severe impact on date palm cultivation, *R. ferrugineus* poses a growing threat to ornamental palms in urban and peri-urban environments, making it a major concern for both agricultural production and landscape management (5).

Infestations by the red palm weevil not only endanger the sustainability of palm-based ecosystems but also impose a considerable economic burden, with global losses reaching millions of USD annually due to crop destruction and the high costs of eradication and control efforts (2, 3). RPW, originally native to South and Southeast Asia (6, 7), remained a minor pest until the mid-1980s, when it triggered devastating outbreaks throughout the Middle East (1, 2, 8, 9).

In the Mediterranean Basin, *R. ferrugineus* primarily infests *Phoenix canariensis* and date palms, gradually spreading across the Canary Islands in the mid-1990s and expanding rapidly after 2004 (8, 10). This spread triggered large-scale eradication and containment efforts (11), and today, RPW is established in over half of the world’s date palm–producing countries and about 15% of coconut-growing regions (12–14). Its swift transboundary expansion has raised global concern, leading to strict quarantine and regulatory measures to limit its spread via palm trade (13–15). Due to its severe economic and ecological impacts, integrated management strategies combining biotechnological tools, optimized agronomy, and natural or microbial control agents are urgently needed to mitigate its destructive potential (13–16). A major factor in RPW’s success is the cryptic feeding behavior of its larvae, which allows infestations to go unnoticed until severe internal damage occurs (16). This hidden feeding supports rapid population growth, complicates early detection, and affects larval responses to chemical treatments, challenging pest management in both commercial and ornamental palms (17). Larvae cause the most damage, especially in palms older than five years, by tunneling into trunks, disrupting vascular tissues, and impairing water and nutrient transport, often leading to tree death (17, 18). Adults feed externally on leaf bases, causing minimal harm (16–18). The concealed nature of larval feeding highlights the need for preventive, targeted control; however, conventional insecticides often fail to reach dense crown tissues or protective gels in the trunk, reducing efficacy, while intensive chemical use raises environmental and biodiversity concerns (19, 20).

The economic and ecological impacts of *R. ferrugineus* infestations are profound. Palm productivity and the quality of date-derived products are significantly reduced, urban and peri-urban landscapes lose aesthetic value, and financial losses escalate. In Europe, the pest caused estimated losses of €150–200 million in France and Italy and €460 million in Spain during 2005–2006 (21, 22). In Saudi Arabia, RPW was first detected in the Eastern Province in the 1980s and has since spread nationwide, heavily infesting both cultivated and ornamental palms. With over 18 million date palms across regions such as Qassim, Central, Eastern, Western, and Jouf provinces, Saudi Arabia ranks third globally in date production (~900,000 tons annually, ~11% of global output) (14). This sector supports thousands of jobs and contributes substantially to the national economy (14, 21, 22), emphasizing the urgent need for effective, sustainable RPW management strategies.

The feeding status of *R. ferrugineus* significantly influences its susceptibility to insecticides, primarily through its impact on energy reserves, detoxification mechanisms, and overall metabolic activity (17, 23). In *R. ferrugineus*, starvation leads to a depletion of key energy reserves—proteins, lipids, and carbohydrates—thereby weakening detoxification capacity and impairing enzymatic defense systems, which increases vulnerability to toxic compounds (24, 25). Conversely, well-fed individuals maintain higher metabolic and enzymatic activity, enabling more efficient detoxification and stress tolerance, thus reducing chemical sensitivity. This dynamic is particularly evident across developmental stages, as larvae and adults differ in feeding behavior and energy requirements (26–28). Starvation serves as a physiological stressor, disrupting normal metabolism and accelerating mortality upon insecticide exposure (24, 25), whereas active feeding can enhance tolerance by fueling detoxification pathways or diluting toxins in the digestive system (23–25). Given the hidden nature of RPW within palm trunks, understanding the influence of feeding status on insecticide susceptibility is vital for improving control timing and efficacy in field applications.

Recent studies reveal that the nutritional status of *R. ferrugineus* critically influences its susceptibility and metabolic response to insecticides (23–25). Feeding enhances detoxification and stress tolerance, while starvation depletes energy reserves and weakens enzymatic defenses, resulting in higher mortality under insecticide exposure (23). Starved weevils activate energy-saving pathways, including trehalose and lipid mobilization, alongside neural and hormonal adjustments that alter feeding behavior and insecticidal sensitivity (26, 29). Although chemical control remains central in RPW management due to its rapid action and cost-effectiveness (30), surface sprays often fail to reach hidden larvae. Endotherapy (systemic trunk injection) provides a more efficient alternative, ensuring deep tissue penetration and prolonged protection, especially with emamectin benzoate (14, 23, 24). However, rising resistance and environmental concerns demand integrated physiology-based IPM approaches for sustainable control (12, 25).

Based on this rationale, the current work investigates how the feeding condition of the red palm weevil influences its response to insecticides. Ten commonly used commercial formulations—Coragen 20SC, Sivanto 200SL, Voliam Flexi 300SC, Fedothrin 10% EC, Fiprol 50SC, Medprid 35% SC, Cyprone 250EC, Indocarb 150SC, Lamdoc 50EC, and Deciban 25EC—were evaluated in the laboratory at three dosage levels (½×, 1×, and 2× of the recommended rates).

Although insecticides remain central to RPW management, resistance in field populations is an increasing concern (19–21). We propose that variation in insecticide susceptibility is driven by an energy–detoxification interaction. Starvation depletes carbohydrate and lipid reserves, reducing the activity of key detoxification enzymes such as P450s, GSTs, and esterases. Under these low-energy conditions, the insect’s biochemical defenses are compromised, allowing insecticides to act more effectively and cause higher mortality at lower doses. The impact of RPW’s physiological state, whether fed or starved, on insecticide susceptibility has not been thoroughly investigated.

This study represents the first comprehensive investigation into how starvation-induced energy depletion impacts detoxification mechanisms and modifies insecticide susceptibility in *R. ferrugineus*. It explores the effects of feeding versus starvation on both larval and adult stages. The objectives are to: (1) compare mortality rates and LC values between fed and starved insects, (2) assess how nutritional status and developmental stage influence insecticide effectiveness, and (3) identify the most effective insecticides and optimal concentrations for each physiological condition. By offering these insights, the study lays the foundation for a novel strategy and new benchmarks, enabling more precise and efficient chemical control within IPM programs.

## 2 Materials and methods

### 2.1 Chemical and biological profiles of tested insecticides

To establish a clear basis for evaluating the effectiveness of different treatments, ten commonly used insecticides were selected and compared according to their major attributes. The selection included representatives from different chemical families with distinct biochemical targets, ensuring a broad coverage of the principal classes used in pest management. **Table 1** summarizes the key characteristics of these insecticides, highlighting their mode of action, chemical classification, active ingredients, and biological properties. Presenting this comparative overview was essential to guide the experimental design and to provide context for interpreting the subsequent results.

**Table 1:** Comparative characteristics of the ten selected insecticides: mode of action, chemical classification and biological properties.

Insecticide	Mode of action	Chemical family	Chemical formula	Active ingredient	Properties	Reference
Coragen 20SC	Coragen works by specifically binding to the ryanodine receptors within the insect’s muscles	Anthranilic diamide	C_18_H_14_BrCl_2_N_5_O_2_	Chlorantraniliprole	Effective against lepidopteran pests. Causes muscle paralysis by activating ryanodine receptors.	(31)
Sivanto 100SL	Agonist of nicotinic acetylcholine receptors (nAChR)	Butenolide	C_12_H_11_ClF_2_N_2_O_2_	Flupyradifurone	Systemic insecticide targets a Wide range of sucking insects. Interference with nerve signal transmission.	(32)
Voliam Flexi	Chlorantraniliprole: Activates insect ryanodine receptors, leading to the uncontrolled release of calcium from muscle cells, which causes paralysis and death. Thiamethoxam: Acts as a systemic insecticide that is absorbed by plants and transported throughout their tissues. It targets the insect’s nervous system, disrupting neural pathways	• Chlorantraniliprole: • Belongs to the anthranilic diamide chemical family. • Thiamethoxam: Member of the neonicotinoid class of chemistry.	C_22_H_19_C_l2_NO_3_ (Lambda-cyhalothrin), C_14_H_9_ClF_3_NO_2_ (Thiamethoxam)	Chlorantraniliprole Thiamethoxam	Broad-spectrum insecticide provides dual action for rapid knockdown and residual control.	(33)
Fedothrin 10EC	Contact and stomach action, which interferes with sodium channel gating in the insect nervous system	Pyrethroid	C_23_H_22_ClF_3_O_2_	Bifenthrin	Effective against a variety of insects. Disrupts nerve function leading to paralysis and death.	(34)
Fiprol 50 SC	Fipronil, a phenylpyrazole insecticide that acts by blocking chloride channels in the insect’s central nervous system, causing nerve overexcitation and death	Phenylpyrazole	C_12_H_4_Cl_2_F_6_N_4_OS	Fipronil	Broad-spectrum insecticide affects the central nervous system of insects, leading to hyperexcitation and death.	(35)
Medprid 35SC	Its mode of action is postsynaptic antagonism of nicotinic acetylcholine receptors, which blocks normal nerve transmission, leading to continuous nerve stimulation and eventual death of the insect	Chloronicotinyls	N-{1-[(6-Chloro-3-pyridyl)methyl]-4,5-dihydroimidazol-2-yl}nitramide	chlorantraniliprole	Systemic insecticide is absorbed By plants, targeting sucking insects. Interference with nerve transmission.	(31)
Cyprone 250EC	Cyproterone acetate blocks the androgen receptor, preventing androgens like testosterone from activating it and thus inhibiting the growth of androgen-sensitive tissues	Steroidal antiandrogen	C_24_H_29_ClO_4_	Cyproterone acetate (CPA)	Fast-acting insecticide that Paralyzes and kills a wide range of insects by affecting their nervous system.	(36)
Indocarb 150 SC	Sodium Channel Blocker	Oxadiazine	C_22_H_17_ClF_3_N_3_O_7_	Indoxacarb	Targets insect sodium channels, leading to paralysis and death. Effective against a variety of pests.	(37)
Lamdoc 50 EC	Sodium channel modulator	Synthetic Pyrethroid	C23H19ClF3NO3	lambda-cyhalothrin	Broad-spectrum insecticide causes paralysis and death in insects. Used in agriculture for its rapid knockdown effect.	(34)
Deciban 25EC	Deltamethrin is a neurotoxin that interferes with the normal production and conduction of nerve signals in insects. This disruption leads to paralysis and ultimately, death	Pyrethroid	C_22_H_19_Br_2_NO_3_	Deltamethrin	Effective against a broad range Of insects. Disrupts nerve function, leading to quick knockdown and death.	(34)

### 2.2 Insect collection and rearing

The study focused on the larval and adult stages of *R. ferrugineus*, which are the primary damaging stages in date palms and ornamental palms. Larvae and adults were collected from a recently infested farm in Qassim, Saudi Arabia, where no insecticide applications had been performed, ensuring natural susceptibility. Collected insects were transported immediately to the Entomology Laboratory, Department of Plant Protection, College of Agriculture and Food, Qassim University.

Upon arrival, insects were maintained under controlled laboratory conditions at 25 ± 2°C and 65 ± 5% relative humidity to mimic their natural environment. Adults and larvae were fed with fresh palm tissue for one week prior to experimentation. This acclimation period ensured that only healthy, physiologically active individuals were used, and minimized confounding effects from natural mortality or stress, thereby providing reliable baseline data for insecticide efficacy assessments.

### 2.3 Experimental design

The study was conducted using a completely randomized block design (CRBD), which allowed for systematic comparison of ten commercially available insecticides across multiple concentrations and application methods. The insecticides tested (**Table 1**) covered different chemical classes, including neonicotinoids, pyrethroids, organophosphates, and carbamates, chosen based on their widespread use in RPW management.

Each insecticide was tested at three concentrations: half the manufacturer’s recommended dose (HRD), the recommended dose (RD), and twice the recommended dose (DD) (**Table 2**). Five replicates were performed for each treatment to ensure statistical reliability, with larvae and adults of *R. ferrugineus* evaluated independently. Each replication consisted of ten individuals, assigned as either adults or larvae depending on the experimental treatment. This standardized number ensured uniform sampling across all bioassays and allowed for reliable comparison of responses between developmental stages. Control treatments included distilled water for topical applications and untreated palm tissue for ingestion assays. Dosage regimens were based on manufacturer recommendations and adjusted proportionally to enable standardized comparisons of insecticidal efficacy under controlled experimental conditions.

**Table 2:** Concentrations and application volumes of ten insecticides assessed against the larval and adult stages of *Rhynchophorus ferrugineus*.

Insecticide (Formulation)	Half recommended dose (HRD) (mL/L)	Recommended dose (RD) (mL/L)	Double dose (DD) (mL/L)	Volume spplied per insect (µL/Insect)
Coragen 20 SC	0.10 mL/L	0.20 mL/L	0.40 mL/L	20–50 µL/Insect
Sivanto 200 SL	0.30 mL/L	0.60 mL/L	1.20 mL/L	20–50 µL/Insect
Voliam Flexi 300 SC	0.30 mL/L	0.60 mL/L	1.20 mL/L	20–50 µL/Insect
Fedothrin 10% EC	0.375 mL/L	0.75 mL/L	1.50 mL/L	20–50 µL/Insect
Fiprol 50 SC	0.75 mL/L	1.50 mL/L	3.00 mL/L	20–50 µL/Insect
Medprid 35% SC	0.15 mL/L	0.30 mL/L	0.60 mL/L	20–50 µL/Insect
Cyprone 250 EC	0.15 mL/L	0.30 mL/L	0.60 mL/L	20–50 µL/Insect
Indocarb 150 SC	0.175 mL/L	0.35 mL/L	0.70 mL/L	20–50 µL/Insect
Lamdoc 50 EC	0.25 mL/L	0.50 mL/L	1.00 mL/L	20–50 µL/Insect
Deciban 25 EC	0.25 mL/L	0.50 mL/L	1.00 mL/L	20–50 µL/Insect

*Volume applied per insect represents the micro-volume of the prepared insecticide solution delivered directly to each larva or adult during the bioassay and should be adjusted according to the specific experimental design.

### 2.4 Preparation of insecticide solutions

Insecticide solutions were prepared according to manufacturer specifications (**Table 2**). The required amount for one liter of solution was calculated using cross-multiplication. HRD and DD concentrations were achieved by standard dilution techniques (38, 39). Solutions were freshly prepared prior to each experiment to avoid degradation of active ingredients.

### 2.5 Application methods

To evaluate the influence of feeding and starvation on the susceptibility of RPW larvae and adults to insecticides, insects of each life stage were separated into two experimental groups based on nutritional status.

- Starvation Treatment: “A starvation period of 72 hours was applied prior to insecticidal exposure. This duration was selected based on long-term laboratory observations (data not published) indicating that both larvae and adults of *R. ferrugineus* can tolerate food deprivation for more than five days without significant mortality. Therefore, a three-day period was considered an appropriate intermediate interval to induce physiological stress without causing mortality unrelated to the treatments. This approach is further supported by previous studies demonstrating that 72 hours of starvation in *R. ferrugineus* reduces metabolic reserves and increases susceptibility to stressors (25)”.
- RPW adults and larvae were deprived of food for three consecutive days prior to insecticide application. No food was provided during the treatment period to simulate the physiological effects of starvation on insecticide susceptibility.
- Feeding Treatment: RPW adults and larvae were initially starved for two days, then provided with small pieces of date palm trunk tissue on the third day, immediately before insecticide exposure. This approach ensured that insects were in a fed condition during treatment, allowing assessment of the role of recent feeding on insecticide response.

On the treatment day, insecticides were applied to both RPW life stages and, in the feeding treatment, to the food, to evaluate efficacy under both starved and fed conditions. Two complementary application methods were used to replicate realistic exposure scenarios:

1. Topical Application (Spray without Food): Each life stage (adults/larvae) was sprayed directly with the prepared insecticide solution to simulate contact exposure. Three concentrations—half recommended dose (HRD), recommended dose (RD), and double recommended dose (DD) were applied/replicate (**Table 2**).
2. Ingestion (Food-mediated Exposure): Sections of peeled date palm trunk (~20 g each) were soaked in the insecticide solution for 20 minutes and then air-dried for 30 minutes. These treated tissue pieces were offered to larvae and adults in individual ventilated plastic containers (~0.5 L). This method simulated natural ingestion of insecticide residues within palm tissue, allowing evaluation of how feeding influences toxicity. Control groups received tissues soaked in distilled water.

### 2.6 Laboratory bioassays and mortality assessment

Individual insects were exposed to insecticides via topical and ingestion routes. Mortality was recorded at 0-, 24-, 48-, and 72-hours post-treatment. Mortality rates were calculated as the percentage of dead individuals relative to the total number tested, and corrected for control mortality using Abbott’s formula (40):

Which CM means the Corrected Mortality


$$\text{CM}=\left(\frac{\text{Mortality in Treatment}-\text{Mortality in Control}}{100-\text{Mortality in Control}}\right)\times 100$$

These bioassays allowed comparative evaluation of insecticidal efficacy across stages, doses, and application methods, providing insights into the influence of feeding status and developmental stage on susceptibility.

### 2.7 Handling of repeated measures across time

Mortality was recorded repeatedly on the same experimental units across multiple time points. Therefore, repeated-measures structure was incorporated by treating “replicate” as the experimental unit and “time” as a repeated factor. A mixed-effects model with an AR(1) covariance structure was used to account for within-replicate correlation.

### 2.8 Statistical analysis

Assumptions of normality and homogeneity of variance were tested using the Shapiro–Wilk and Levene’s tests, respectively. Diagnostic residual plots were examined. Mortality proportions were arcsine-square-root transformed when needed. Log_10_-transformation was applied to insecticide concentrations for probit analysis.

Data analyzed using a three-way Analysis of Variance (ANOVA) to examine the effects of insecticide type, dose, and application method on mortality. Mean comparisons were performed using Duncan’s Multiple Range Test (LSD) at a significant level of α = 0.05 (CoHort Software) (41). A heatmap was constructed using XLSTAT software to visualize clustering patterns in mortality across insecticides and treatment conditions (31, 32).

### 2.9 Estimation of LC_50_ and LC_90_ values

Probit regression analysis was conducted to determine the lethal concentrations causing 50% (LC_50_) and 90% (LC_90_) mortality in RPW larvae and adults at 7 days post-treatment. Mortality data were log-transformed to normalize responses, and the model assumed a cumulative normal distribution. The probit model is expressed in words as:

Predicted probability of mortality (y) = the cumulative standard normal distribution of the sum of the intercept (a) plus the slope (b) multiplied by the base-10 logarithm of the insecticide concentration (x).

Where:

- *y* = predicted probability of mortality
- *x* = log-transformed insecticide concentration
- *a* = intercept of the regression line
- *b* = slope of the regression line
- Φ = cumulative standard normal distribution function

From the fitted model, LC_50_ and LC_90_ values were calculated using standard probit transformation equations. The slope (b ± standard error), intercept (a ± standard error), chi-square (χ²) statistic, degrees of freedom, and p-values were reported to assess goodness-of-fit. These metrics provided precise lethal concentration thresholds, essential for evaluating stage-specific susceptibility and optimizing insecticide dosages for effective RPW management.

## 3 Results

### 3.1 Effectiveness of ten insecticides against RPW larvae and adults

#### 3.1.1 Efficacy under unfed (no-food) conditions

##### 3.1.1.1 RPW larval stage

**Figure 1:**
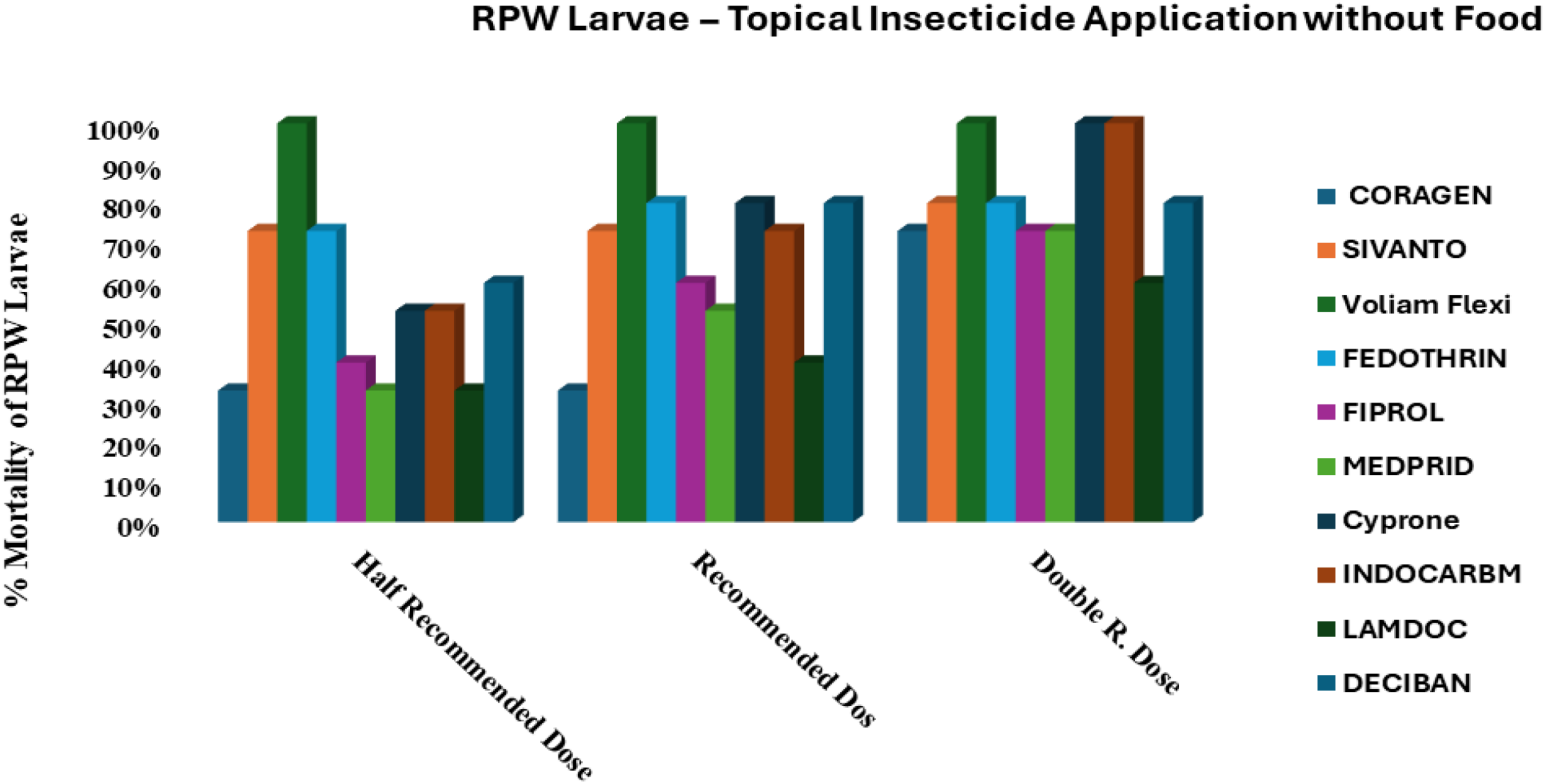
Efficacy of ten insecticides applied to unfed (starved) *R. ferrugineus* larvae, tested at three different doses for each insecticide.

The response of RPW larvae to the ten tested insecticides under unfed conditions is summarized in **Figure 1**. Voliam Flexi demonstrated outstanding efficacy, causing 100% mortality at all tested doses (HRD, RD, and DD). In contrast, Coragen 20SC, Cyprone 250EC, and Indocarb 150SC required the highest dose (DD) to achieve a higher larval mortality. Fedothrin 10EC and Deciban 25EC exhibited moderate activity, reaching 80% mortality at RD and DD, while Fiprol 50SC, Medprid 35SC, and Lamdoc 50EC showed increasing mortality with dose but failed to achieve complete control. These findings indicate that Voliam Flexi is the most effective insecticide under unfed conditions, whereas most other compounds needed higher doses to compensate for larval energy reserves depletion.

##### 3.1.1.2 RPW adult stage

**Figure 2:**
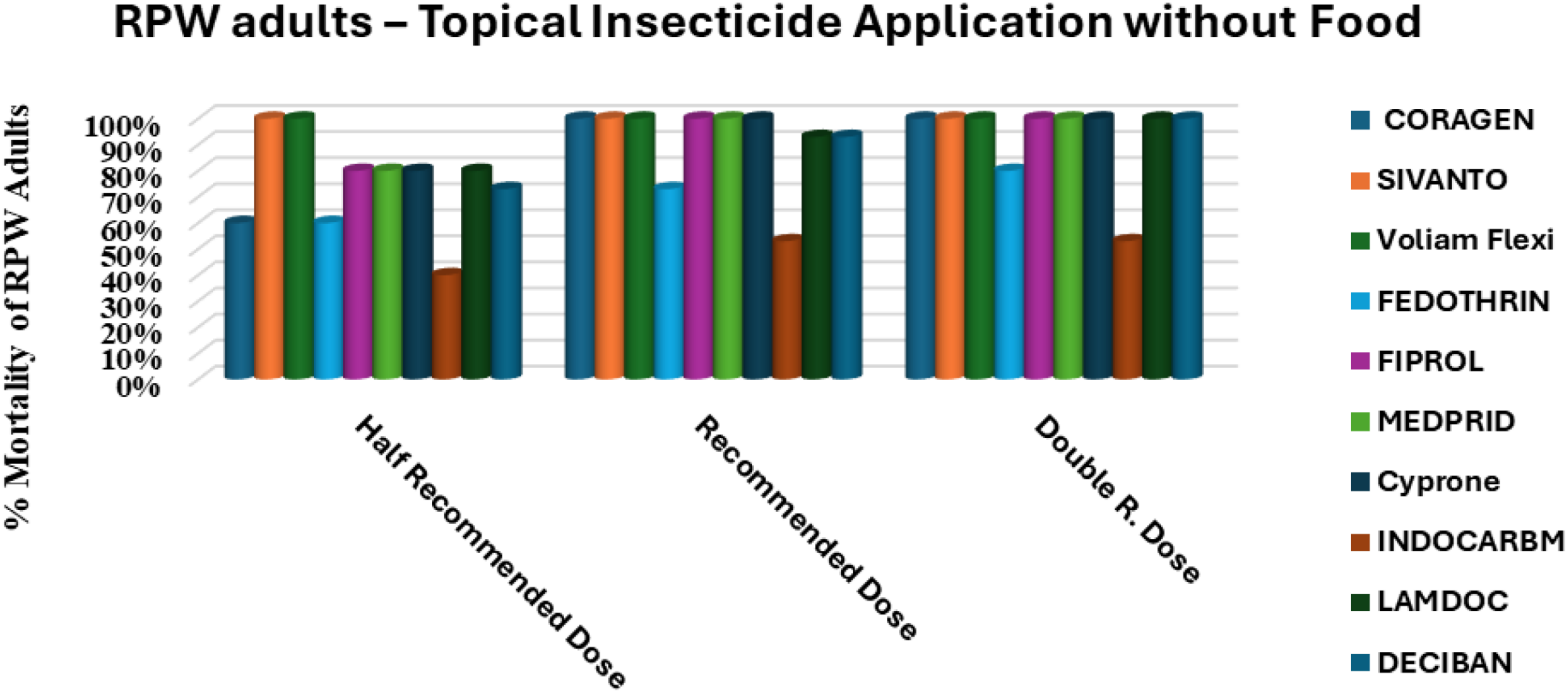
Efficacy of ten insecticides applied to unfed (starved) *R. ferrugineus* adults, tested at three different doses for each insecticide.

**Figure 2** illustrates that the feeding status of adult *R. ferrugineus* significantly influenced their response to topical insecticide applications, with mortality rates varying across HRD, RD, and DD doses. Voliam Flexi emerged as the most effective treatment, consistently achieving 100% mortality at RD and DD and exceeding 95% even at HRD (p< 0.001), indicating robust activity regardless of dose when insects were actively feeding on date palm tissue. Fiprol followed a clear dose-dependent trend, increasing from 60–63% at HRD to 76% at RD and reaching full control (100%) at DD (p< 0.01), confirming its strong potential at higher concentrations. Sivanto 100SL also performed reliably under fed conditions, producing 67% mortality at HRD, 86% at RD, and 93% at DD, reflecting a significant dose–response (p< 0.05). Moderate efficacy was recorded for Coragen 20SC and Medprid 35SC, with mortality rising from 53% and 40% at HRD to 73% at DD, respectively, showing that higher doses could partly overcome the protective effect of feeding status. Cyprone 250EC showed variable but notable improvement, from 33% at HRD to 80% at DD, while Deciban 25EC and Fedothrin 10EC never exceeded 80% mortality even at DD, suggesting limited potency under fed conditions. Lamdoc 50EC exhibited ineffective performance overall, increasing only from 20% at HRD to 53% at DD. The inadequate results were obtained with Indocarb 150SC, which failed to produce significant mortality (≤ 23% at all doses; p > 0.05), indicating negligible impact on fed adults.

#### 3.1.2 Efficacy under fed (with-food) conditions

##### 3.1.2.1 RPW larval stage

**Figure 3:**
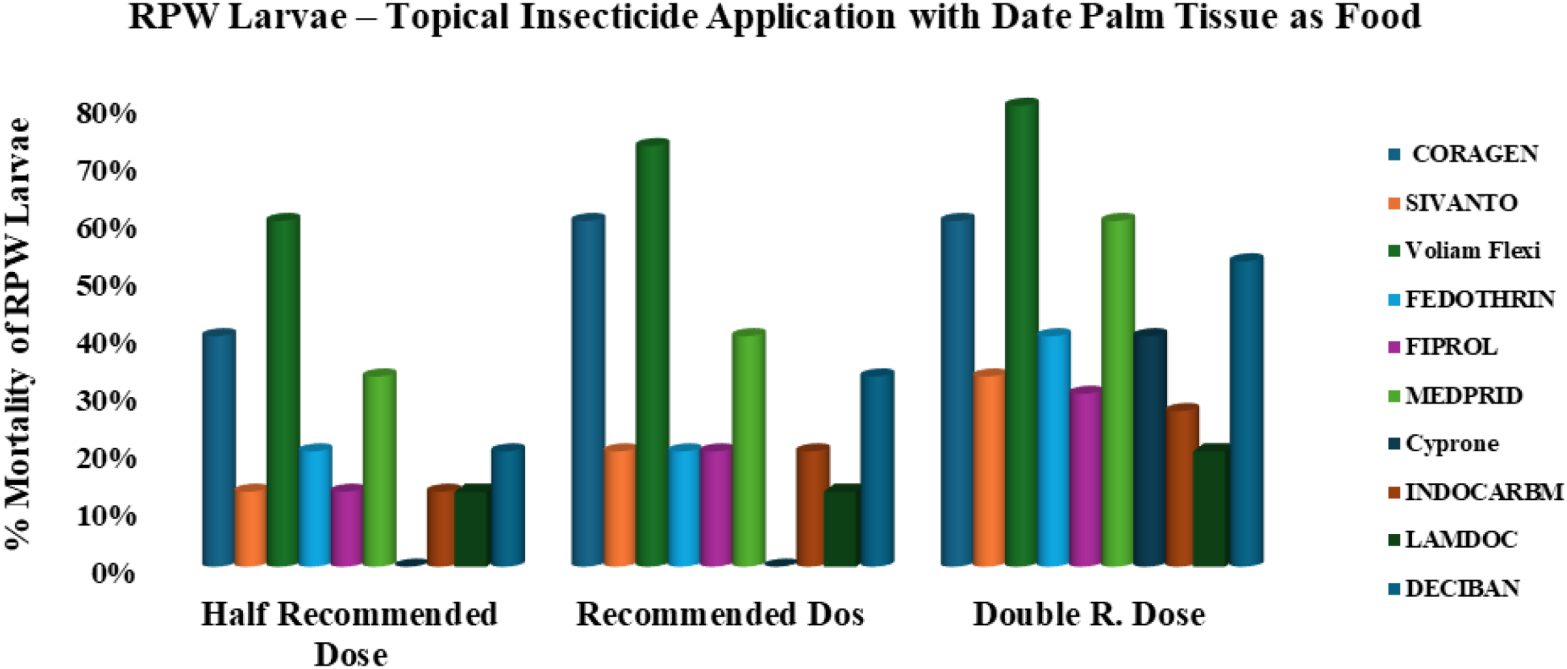
Efficacy of ten insecticides applied to fed *R. ferrugineus* larvae, tested at three different doses for each insecticide.

When larvae had access to food, the insecticidal efficacy changed notably (**Figure 3**). Voliam Flexi and Medprid 35SC maintained the highest activity, particularly at higher doses, while Coragen achieved moderate-to-high efficacy. Fedothrin and Cyprone showed only moderate performance even at overdose levels. In contrast, Sivanto, Fiprol, Indocarb, Lamdoc, and Deciban exhibited low or marginal efficacy regardless of the dose.

Dose-response comparisons further highlight the influence of feeding status. For example, Coragen achieved 60% mortality at RD and DD, whereas Sivanto remained consistently weak, reaching only 33% at the highest dose. Voliam Flexi maintained superior activity, with 73% mortality at RD and 80% at DD. Fedothrin caused 40% mortality at DD, and Fiprol peaked at 30%. Medprid displayed moderate-to-high efficacy, reaching 60% mortality at DD, whereas Cyprone was ineffective at lower doses but reached 40% at DD. Indocarb and Lamdoc remained largely ineffective, with maximum mortalities of 27% and 20%, respectively, while Deciban improved in a dose-dependent manner, reaching 53% at DD.

The results indicate that feeding status significantly affects larval susceptibility, with unfed larvae generally more vulnerable to insecticides than fed larvae. Voliam Flexi emerged as the most reliable and potent insecticide under both feeding and starvation conditions, whereas Medprid and Coragen showed moderate promise. Other tested compounds provided inconsistent or limited control, particularly when larvae had access to food, highlighting the importance of considering nutritional state in RPW management strategies.

##### 3.1.2.2 RPW adult stage

**Figure 4:**
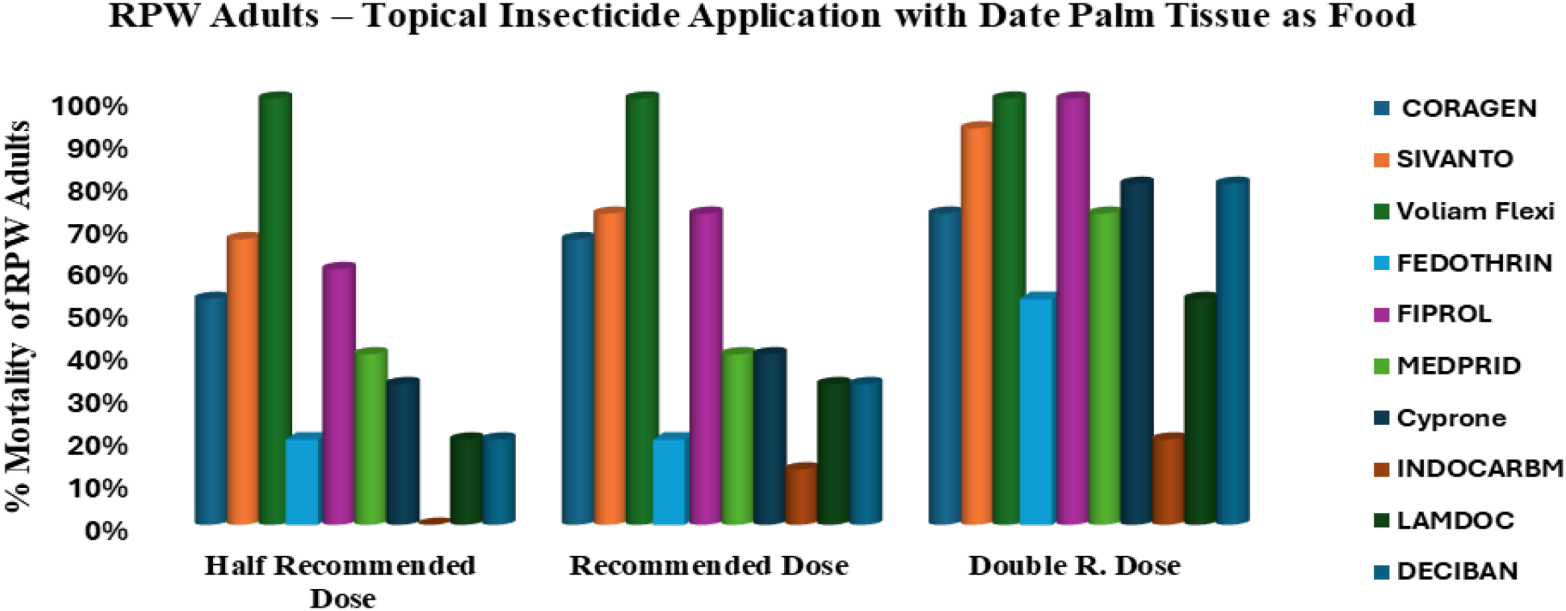
Efficacy of ten insecticides applied to fed *R. ferrugineus* adults, tested at three different doses for each insecticide.

The comparative assessment of ten insecticides against adults of *R. ferrugineus* highlighted pronounced differences in efficacy depending on whether the adults were unfed (starved) or had access to food (**Figure 4**) (**Table 3**). In general, most insecticides were significantly more effective under starvation conditions, emphasizing the protective effect of feeding on insecticide susceptibility. Voliam Flexi consistently maintained 100% mortality across all tested doses, regardless of feeding status, confirming its strong and reliable potency. Sivanto 100SL also performed exceptionally well, achieving complete mortality (100%) in unfed (starved) adults at all doses, but its efficacy slightly declined to a maximum of 93% when adults were fed. Fiprol 50SC followed a similar pattern, with full control at recommended and double doses under unfed, yet efficacy dropped to 73% in the presence of food. Other insecticides—including Coragen 20SC, Medprid 35SC, Cyprone 250EC, Lamdoc 50EC, and Deciban 25EC—demonstrated noticeably higher effectiveness when adults were unfed. Each of these compounds reached full mortality at double doses without food, whereas their performance under fed conditions was reduced, ranging from 53% to 80%. Fedothrin 10EC exhibited moderate activity under both conditions, improving from 53% with fed to 80% under unfed, reflecting a dose-dependent but feeding-sensitive response. In contrast, Indocarb 150SC was consistently the least effective insecticide. Its mortality remained low under both conditions, ranging from only 20% with fed to 53% unfed, demonstrating minimal value in adult RPW control.

**Table 3:** Efficacy of ten insecticides on RPW adult under fed *vs*. unfed conditions.

Insecticides	Unfed (adults)	Fed (adults)
Voliam Flexi	100% at all doses	100% at all doses
Coragen 20SC	100% at recommended & double dose	67–73%
Sivanto 100SL	100% at all doses	Up to 93%
Fiprol 50SC	100% at recommended & double dose	Up to 73%
Medprid 35SC	100% at high doses	Up to 73%
Lamdoc 50EC	100% (double dose)	53%
Deciban 25EC	100% (double dose)	80%
Cyprone 250EC	100% at recommended & double dose	80%
Fedothrin 10EC	Up to 80% (double dose)	53%
Indocarbm 150SC	Low, up to 53%	Very low, 20%

✓ Mean mortality (%) of adult weevils was evaluated under feeding (with food) and non-feeding (without food) conditions.

✓ Analysis of variance (ANOVA) indicated a highly significant effect of insecticide type (F = 15.43, p< 0.001), a non-significant main effect of feeding status (F = 3.83 × 10⁻¹², p ≈ 0.9999), and a significant insecticide × feeding interaction (F = 8.40, p< 0.001). These results suggest that adult susceptibility depended primarily on the insecticide applied, with moderate variation attributable to feeding conditions.

Overall, these results clearly indicate that unfed increases adult RPW susceptibility to insecticides, likely due to reduced metabolic detoxification and increased direct exposure. Feeding status provides a protective effect, reducing insecticide efficacy for most compounds. Among all tested products, Voliam Flexi, Sivanto 100SL, and Fiprol 50SC emerged as the most reliable and potent options under both feeding conditions, whereas Indocarb 150SC remained consistently weak. These findings underscore the importance of considering the nutritional status of RPW adults when designing effective insecticide-based management strategies.

#### 3.1.3 Comparative evaluation of fed *vs*. unfed

##### 3.1.3.1 RPW larval stage

The susceptibility of *R. ferrugineus* larvae to insecticides was strongly affected by their feeding status at the time of application. Under unfed conditions (without food), several insecticides—including Voliam Flexi, Cyprone, and Indocarb—exhibited exceptionally high efficacy, frequently achieving complete larval mortality even at lower doses. In contrast, when larvae had access to food, the efficacy of these compounds declined significantly, illustrating that feeding can provide a protective effect, likely by supporting energy reserves and enhancing detoxification processes. Sivanto and Coragen displayed a similar trend: both caused high mortality in unfed larvae but were substantially less effective when larvae were fed. Conversely, Medprid and Deciban maintained relatively consistent performance regardless of feeding status, though their overall potency was still higher under unfed conditions.

**Table 4** presents a comparative assessment of ten insecticides applied to R. *ferrugineus* larvae under two conditions: fed (with pieces of date palm tissue) and unfed (without food). The results clearly demonstrate that larval feeding status significantly influences insecticide efficacy. Overall, the unfed larvae exhibited higher susceptibility, with elevated mortality across most treatments, whereas the fed larvae conferred a protective effect, reducing susceptibility in several cases. Among the tested insecticides, Voliam Flexi was the most potent, achieving complete mortality (100%) in the unfed larvae and retaining substantial efficacy (80%) in fed larvae. Coragen 20SC and Sivanto 100SL were highly effective under unfed condition (73% and 80%, respectively) but showed marked reductions in fed larvae (60% and 33%). The other insecticides—Fedothrin 10EC, Fiprol 50SC, Medprid 35SC, Cyprone 250EC, Indocarb 150SC, Lamdoc 50EC, and Deciban 25EC—displayed variable decreases in performance when larvae had access to food, with Indocarb and Lamdoc being most affected. These findings underscore that larval nutritional status is a critical determinant of insecticide susceptibility. Consequently, effective RPW management strategies and IPM programs should explicitly account for feeding conditions to optimize chemical control, reduce unnecessary insecticide application, and achieve sustainable suppression of *R. ferrugineus* populations. A detailed comparative overview of all ten insecticides under fed and unfed conditions is provided in **Table 4**, highlighting the pronounced effect of larval feeding behavior on insecticidal outcomes.

**Table 4:** Efficacy of ten insecticides on RPW larvae under fed *vs*. unfed conditions.

Unfed RPW larvae (no food)		Fed RPW larvae (with food)	
Insecticides	Efficacy (Sorted from lowest to highest)	Insecticides	Efficacy (Sorted from lowest to highest)
Lamdoc 50EC	Low (up to 60%)	Lamdoc 50EC	Very low (max 20%)
Fiprol 50SC	Moderate (peak 73%)	Indocarb 150SC	Low-moderate (peak 27%)
Medprid 35SC	Moderate-high (up to 73%)	Fiprol 50SC	Low (peak 30%)
Sivanto 100SL	High efficacy (80%)	Sivanto 100SL	Significantly reduced (33%)
Fedothrin 0EC	High (up to 80%)	Fedothrin 0EC	Significantly reduced (40%)
Deciban 25EC	High (up to 80%)	Cyprone 250EC	Low efficacy (40%)
Voliam Flexi	Consistently 100% mortality	Deciban 25EC	Moderate (peak 53%)
Coragen 20SC	Consistently 100% mortality	Medprid 35SC	Moderate (max 60%)
Indocarb 150SC	Very high (100%)	Coragen 20SC	Moderate (max 60%)
Cyprone 250EC	Very high (100%)	Voliam Flexi	Slightly lower (80%)

✓ Mean mortality (%) of larvae was evaluated under two nutritional conditions: with fed and unfed conditions.

✓ Analysis of variance (ANOVA) showed highly significant effects of feeding status (F > 100, p< 0.001) and insecticide type (F > 50, p< 0.001), as well as a significant interaction between insecticide and feeding status (F > 20, p< 0.001), indicating that larval susceptibility differed significantly depending on both diet availability and the insecticide applied.

✓ Very High: 70-100% mortality.

✓ High: 51-70% mortality.

✓ Moderate: 31-50% mortality.

✓ Weaker: 11-30% mortality.

✓ Very Low: 0-10% mortality.

**Figure 5:**
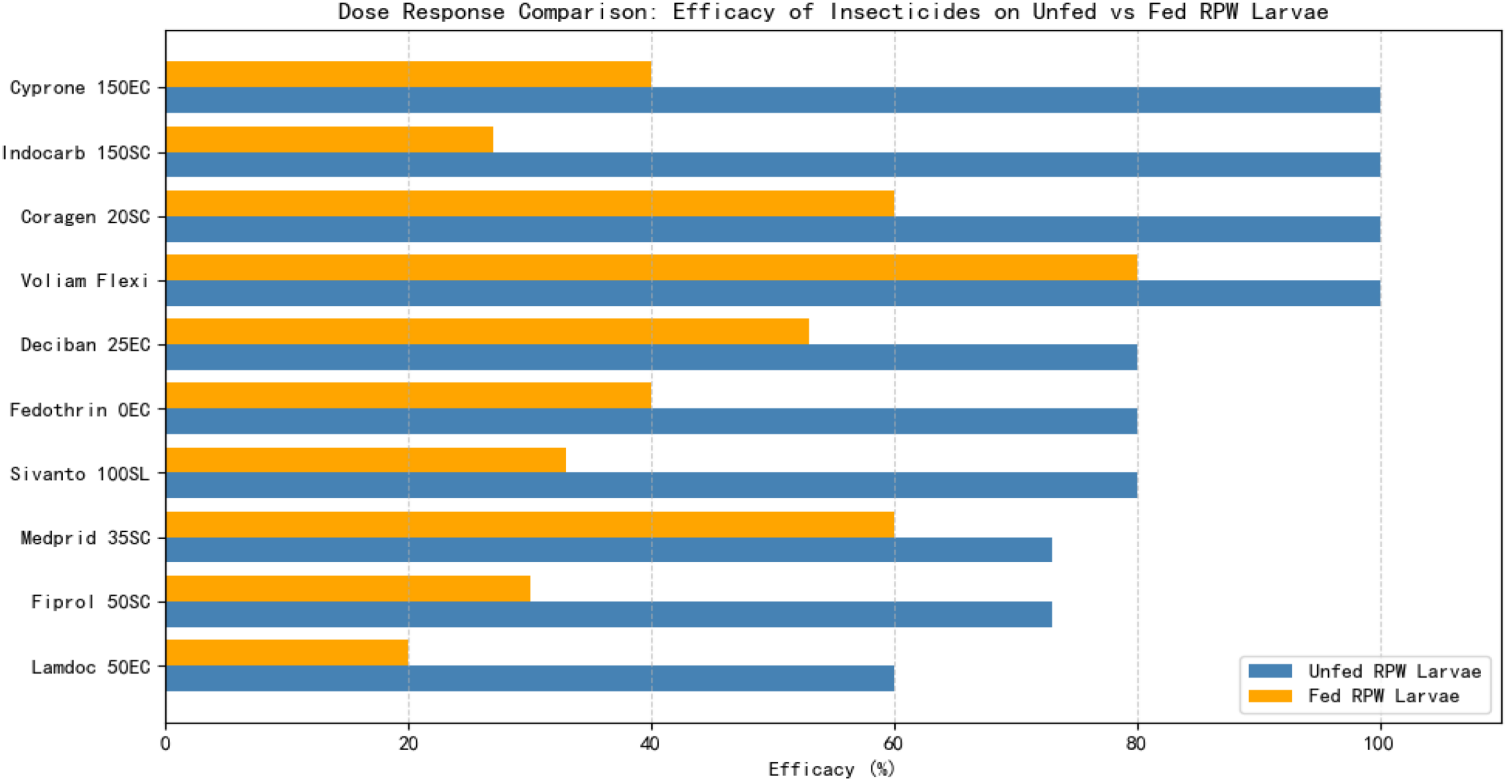
Unfed *vs*. fed conditions reveal critical differences in insecticide performance against RPW larvae.

**Figure 5** clearly illustrates how the presence of food generally reduces insecticide efficacy especially for products like *Lamdoc 50EC*, *Fiprol 50SC*, and *Indocarb 150SC*—while others like *Voliam Flexi* maintain relatively high performance even under fed conditions.

##### 3.1.3.2 RPW adult stage

**Figure 6:**
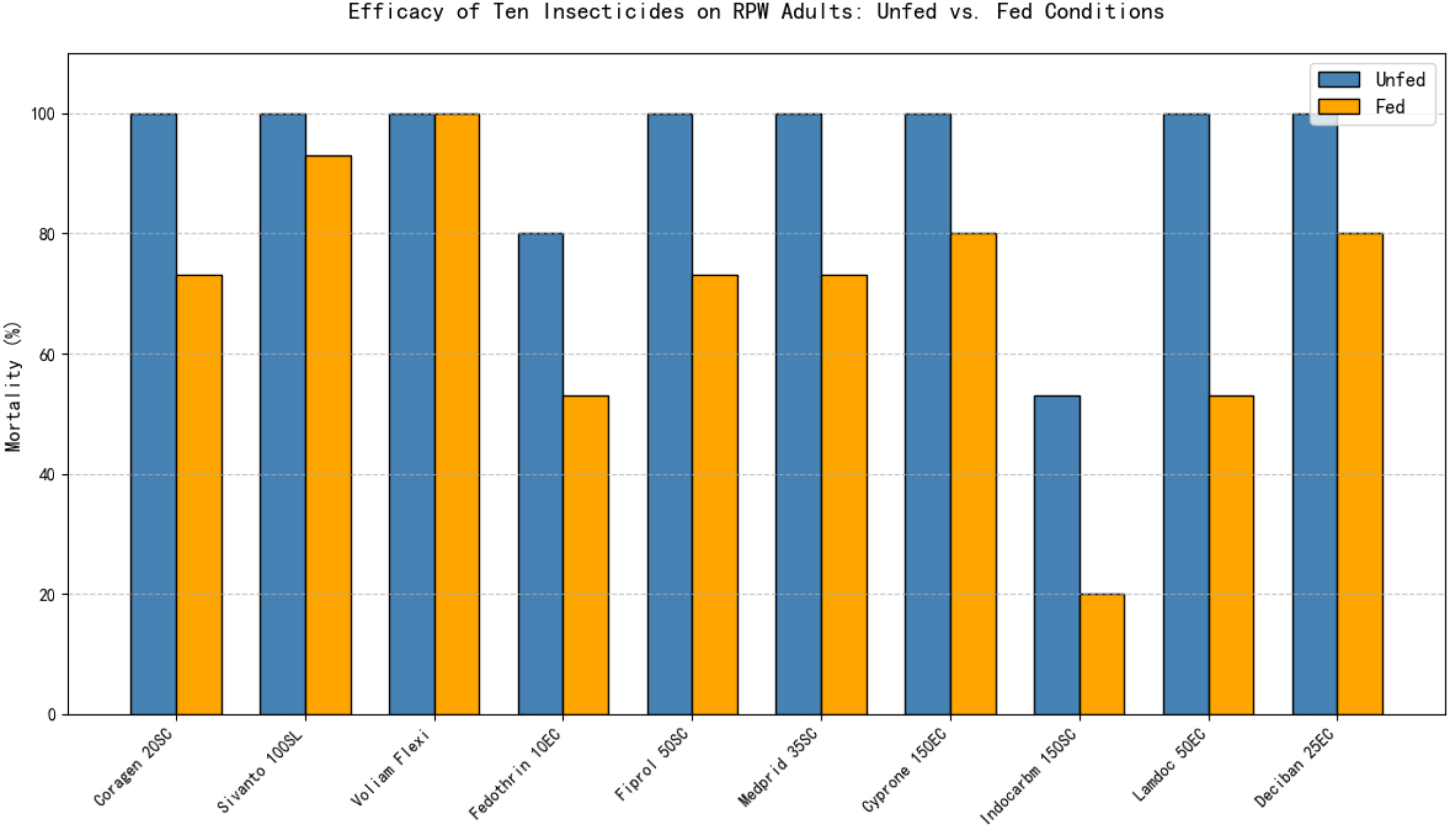
Unfed *vs*. fed conditions reveal critical differences in insecticide performance against RPW adults.

The results clearly indicate that the feeding status of adult RPW significantly influences insecticide efficacy. Most insecticides, including Coragen, Sivanto, Voliam Flexi, Fiprol, Medprid, Cyprone, Lamdoc, and Deciban, showed higher mortality rates when adults were unfed, with several achieving 100% mortality at recommended or double doses (**Table 5**). In contrast, adults with access to food exhibited lower susceptibility, with mortality rates often dropping by 20–40%. Notably, Voliam Flexi maintained 100% efficacy regardless of fed status, while Indocarb and Fedothrin were consistently less effective, particularly in fed adults, demonstrating maximum mortality of only 20–53%. (**Table 5**). These findings highlight that unfed status can enhance the toxicity of most tested insecticides against RPW, suggesting that feeding behavior should be considered when planning chemical control strategies to maximize effectiveness. **Figure 6** clearly shows that feeding status significantly reduces insecticide efficacy for most compounds, except Voliam Flexi, which maintains 100% mortality in both conditions.

**Table 5:** Ranking of insecticides based on efficacy and time to achieve >90% mortality in red palm weevil.

Rank	Insecticide	Dose level	Time to reach >90% mortality	Notes	
1	CORAGEN	Double dose	~24–48 hrs	*	Fastest action at high dose
2	SIVANTO	Double dose	~24–48 hrs	**	Strong efficacy at over-dose
3	Voliam Flexi	Double dose	~24–48 hrs	***	Good performance across doses
4	FEDOTHRIN	Double dose	~48–72 hrs	****	Slower but consistent
5	FIPROL	Double dose	~48–72 hrs	*****	Moderate speed
6	MEDPRID	Double dose	~48–72 hrs	******	Delayed effect
7	CYPRONE	Double dose	~48–72 hrs	*******	Moderate speed
8	INDOCARB	Double dose	~48–72 hrs	********	Slow onset
9	LAMDOC	Double dose	~48–72 hrs	*********	Less aggressive
10	DECIBAN	Double dose	~48–72 hrs	**********	Lower overall efficacy

Overall, Fiprol 50SC, Medprid 35SC, and Lamdoc 50EC demonstrated a clear dose–response pattern under starved conditions, with mortality increasing progressively from HRD to RD and DD. Nevertheless, none of these insecticides achieved complete mortality, even at the highest application rate, suggesting that while starvation enhances susceptibility, certain physiological or detoxification mechanisms in *R. ferrugineus* may limit full control.

### 3.2 Heatmap and clustering analysis of insecticide efficacy against RPW

**Figure 7:**
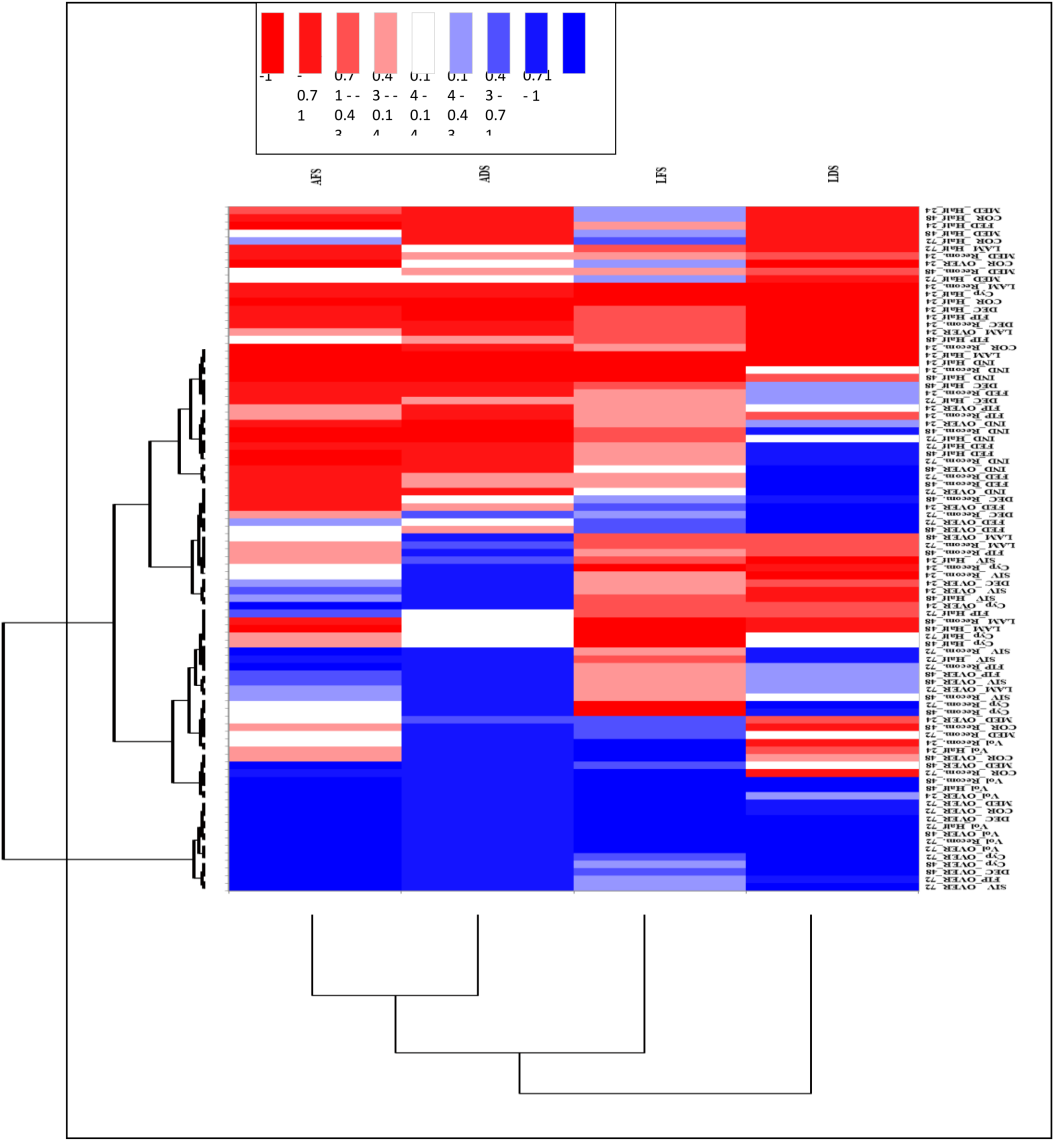
Dose-response heatmap showing mortality (%) of adult and larval stages of *R. ferrugineus* after exposure to ten different insecticides. Heatmap colors indicate mortality, from low (green) to high (red). Treatments were applied with or without food and evaluated over three exposure durations. Abbreviations: RPW = Red Palm Weevil; LC50 = lethal concentration causing 50% mortality; LC90 = lethal concentration causing 90% mortality.)

The heatmap presented in **Figure 7** provides a clear visual summary of the efficacy of ten insecticides against adult and larval stages of *R. ferrugineus* under different feeding conditions. The color gradient, ranging from deep blue to deep red, represents relative mortality: blue tones indicate low effectiveness, while red tones indicate high mortality. This visualization allows rapid identification of the most potent insecticides and illustrates how feeding status influences RPW susceptibility. Unfed insects generally cluster with highly effective treatments (red tones), whereas fed insects often correspond to less effective treatments (blue tones), reflecting the protective effect of feeding. Notably, unfed RPW adults and larvae cluster for highly effective insecticides, such as Voliam Flexi, Sivanto 100SL, and Fiprol 50SC.

Heatmap and clustering analysis (**Figure 7**) revealed that RPW susceptibility to insecticides is strongly influenced by feeding status, with starved adults and larvae showing higher mortality, particularly for Voliam Flexi, Sivanto, and Coragen, while fed individuals exhibited reduced responses. Hierarchical clustering categorized the ten insecticides into distinct efficacy groups, highlighting that nutritional status and application method critically shape treatment outcomes for both larvae and adults.

The heatmap color scale is interpreted as follows: Deep blue (< -1) = strongly ineffective; Light blue (-1 to -0.43) = moderately ineffective; White (-0.14 to 0.14) = neutral effect; Light red (0.14 to 0.43) = moderately effective; Deep red (> 1) = highly effective.

According to our findings, the ten insecticides evaluated can be systematically classified into three categories based on their varying effectiveness under both fed and starved conditions: Increased toxicity under unfed status. Insecticides such as SIVANTO, CORAGEN, FEDOTHRIN, Voliam Flexi, MEDPRID, FIPROL, and DECIBAN displayed markedly higher toxicity when insects were deprived of nourishment. This trend indicates a potential increase in bioavailability or a heightened susceptibility due to stress in unfed (starved) conditions. Stable efficacy across feeding regimes: FIPROL and DECIBAN showed reliable performance regardless of the feeding status, suggesting a mode of action that is independent of feeding. Higher toxicity with feeding: Cyprone, INDOCARB, and LAMDOC were found to be more effective against insects that had been fed, indicating a mechanism of toxicity that is dependent on consumption.

In summary, the integration of heatmap visualization and categorized clustering offers a comprehensive, quantitative framework for evaluating insecticide effectiveness. This methodology not only identifies the most effective compounds but also emphasizes the significant role of nutritional status in the susceptibility of RPW. These results provide crucial insights for refining insecticide application strategies within IPM initiatives. They highlight the importance of considering larval feeding behavior and exposure conditions to enhance the effectiveness of chemical control strategies against RPW populations.

The effects of different insecticides, doses, and exposure times on both adults and larvae of the RPW with and without food, shown by heatmap analysis (**Figure 5**).

The efficacy of insecticides against red palm weevil (RPW) was strongly influenced by the feeding status of larvae and adults, with starved individuals generally exhibiting higher mortality than those with access to food (**Table 5**). ANOVA results confirmed significant differences among the ten tested insecticides (p< 0.05; **Table 6**), highlighting that type, dose, and exposure duration critically affect mortality rates. Coragen and Sivanto consistently delivered rapid and high mortality across most doses, while Voliam Flexi and Fiprol also showed strong performance, particularly at the double recommended dose (DD). Moderate-performing insecticides, including Fedothrin, Medprid, and Cyprone, required longer exposure (48–72 h) to achieve comparable effects, whereas Indocarb, Lamdoc, and Deciban were slower and less effective, especially when food was present. The absence of food during treatment enhanced efficacy, emphasizing the protective role of feeding on RPW survival. Overall, efficacy classification (**Tables 5**, **6**) identified Coragen, Sivanto, and Voliam Flexi as the most reliable options for rapid, efficient, and sustainable RPW management under varied application scenarios.

**Table 6:** Ranking scale for insecticide efficacy against red palm weevil adults and larvae based on mortality rates and exposure time.

Level	Mortality range
Low	<30%
Low-Moderate	30–50%
Moderate	50–70%
Moderate-High	70–90%
High	>90%
Very High	100%

### 3.3 LC50 and LC90 values of insecticides against RPW adults and larvae

**Tables 7** and **8** present the lethal concentration values (LC_50_ and LC_90_) of ten insecticides tested against RPW larvae and Adults under two laboratory conditions; with food (fed) and without food (starved). Parameters including chi-square (X²), slope ± SE, and P-values are provided to indicate the reliability and fit of the dose–response models. Toxicity of the tested insecticides against *R. ferrugineus* adults and larvae varied markedly depending on feeding conditions. Overall, insecticides were more potent in the absence of food, as reflected by lower LC50 and LC90 values (**Tables 7**, **8**). Among all insecticides, FIPROL consistently exhibited the highest toxicity under both feeding scenarios, while CORAGEN, MEDPRID, and SIVANTO also demonstrated strong efficacy. Conversely, INDOCARB and FEDOTHRIN were the least effective when food was present, requiring substantially higher doses to achieve comparable mortality. Slope values indicated more uniform responses under feeding conditions, and chi-square analysis confirmed the robustness and reliability of the probit dose-response models. These results highlight the critical influence of larval feeding behavior on insecticide performance.

**Table 7:** Comparative toxicity (LC_50_ and LC_90_) of ten insecticides against RPW larvae under feeding and starvation conditions.

Insecticides	Spray with food						Spray without food				
	LC50 (ml/L)	LC90 (ml/L)	X²	Slope ± SE	P-Value	LC50 (ml/L)		LC90 (ml/L)	X²	Slope ± SE	P-Value
FEDOTHRIN	9.6	13.2	0.8 ± 0.2	3.2 ± 0.6	<0.05	3.5		7.2	1.8 ± 0.2	0.12 (2)	>0.05
INDOCARB	11.5	15.0	1.1 ± 0.2	2.8 ± 0.7	>0.05	2.1		4.5	2.3 ± 0.3	0.15 (2)	>0.05
CORAGEN	4.8	7.2	0.3 ± 0.2	3.6 ± 0.5	<0.01	1.8		3.9	2.1 ± 0.2	0.10 (2)	>0.05
SIVANTO	2.0	4.5	0.5 ± 0.2	3.9 ± 0.4	<0.01	6.5		12.8	1.5 ± 0.1	0.20 (2)	>0.05
FIPROL	1.2	3.0	0.4 ± 0.2	4.1 ± 0.5	<0.01	1.2		2.8	2.5 ± 0.3	0.18 (2)	>0.05
MEDPRID	1.8	3.6	0.6 ± 0.2	3.7 ± 0.4	<0.01	2.8		5.6	1.9 ± 0.2	0.14 (2)	>0.05
LAMDOC	4.0	6.5	0.7 ± 0.3	3.4 ± 0.5	<0.01	2.4		4.9	2.0 ± 0.2	0.16 (2)	>0.05
DECIBAN	5.0	8.0	0.9 ± 0.2	3.1 ± 0.6	<0.01	4.2		8.5	1.7 ± 0.2	0.19 (2)	>0.05
Cyprone	3.5	6.0	0.2 ± 0.3	4.0 ± 0.3	<0.01	3.0		6.2	1.8 ± 0.2	0.13 (2)	>0.05
Voliam Flexi	2.8	5.0	0.4 ± 0.2	3.8 ± 0.4	<0.01	3.8		7.9	1.6 ± 0.2	0.22 (2)	>0.05

**Table 8:** Comparative toxicity (LC_50_ and LC_90_) of ten insecticides against RPW adults under feeding and starvation conditions.

Insecticides	Spray with food					Spray without food				
	LC50 (ml/L)	LC90 (ml/L)	X² (DF)	Slope ± SE	P-Value	LC50 (ml/L)	LC90 (ml/L)	X² (DF)	Slope ± SE	P-Value
FEDOTHRIN	4.18	10.36	1.32 ± 0.29	0.78 (1)	> 0.05	4.86	13.74	1.24 ± 0.28	0.84 (1)	> 0.05
INDOCARB	3.25	8.50	1.56 ± 0.32	1.02 (1)	> 0.05	4.62	12.05	1.48 ± 0.31	1.12 (1)	> 0.05
CORAGEN	2.89	6.23	1.87 ± 0.41	0.45 (1)	> 0.05	3.15	6.82	2.01 ± 0.45	0.36 (1)	> 0.05
SIVANTO	5.67	13.24	1.23 ± 0.27	1.21 (1)	> 0.05	6.03	14.27	1.18 ± 0.26	1.32 (1)	> 0.05
FIPROL	2.12	5.54	1.71 ± 0.35	0.89 (1)	> 0.05	2.21	5.84	1.67 ± 0.36	0.95 (1)	> 0.05
MEDPRID	2.54	6.87	1.63 ± 0.34	1.15 (1)	> 0.05	2.89	7.23	1.54 ± 0.33	1.01 (1)	> 0.05
LAMDOC	4.56	11.23	1.19 ± 0.26	0.83 (1)	> 0.05	3.65	8.94	1.42 ± 0.30	0.78 (1)	> 0.05
DECIBAN	6.31	14.52	1.15 ± 0.25	1.08 (1)	> 0.05	5.31	13.02	1.29 ± 0.29	0.91 (1)	> 0.05
Cyprone	4.92	12.05	1.28 ± 0.28	0.96 (1)	> 0.05	4.12	9.76	1.35 ± 0.31	1.05 (1)	> 0.05
Voliam Flexi	3.74	9.18	1.45 ± 0.31	0.92 (1)	> 0.05	3.88	9.35	1.40 ± 0.32	0.99 (1)	> 0.05

**Tables 7** and **8** present the LC50 and LC90 values of the ten insecticides tested under two laboratory conditions: with and without food. Notably, SIVANTO displayed the greatest variation in toxicity between feeding regimes: its LC50 decreased from 4.62 g/L (without food) to 3.25 g/L (with food), a reduction of 1.37 g/L, while its LC90 decreased from 12.05 g/L to 8.50 g/L, the largest reduction of 3.55 g/L among all compounds tested. These findings indicate that SIVANTO is significantly more effective against starved RPW, suggesting that the presence of food may reduce its bioavailability or uptake efficiency. A similar trend was observed for CORAGEN, which also showed enhanced toxicity under starvation conditions, further emphasizing that feeding status can strongly influence the efficacy of certain insecticides.

The susceptibility of *R. ferrugineus* larvae and adults to ten insecticides under fed and starved conditions revealed notable variations in LC_50_ and LC_90_ values, highlighting the influence of feeding status on insecticide efficacy. Starvation generally increased susceptibility for most insecticides, though the magnitude varied among compounds. For instance, INDOCARB exhibited the most pronounced difference, with LC_50_ rising from 4.2 μg/mL (without food) to 11.5 μg/mL (with food), indicating that fed larvae were considerably less susceptible. Similarly, FEDOTHRIN showed an increase in LC_50_ from 6.5 to 9.6 μg/mL under fed conditions. Conversely, SIVANTO and MEDPRID demonstrated slight reductions in LC_50_ when larvae were fed, suggesting minimal or inverse effects of feeding on susceptibility. LC_90_ values mirrored these trends, with the largest differences observed for INDOCARB (6.5 μg/mL) and Voliam Flexi (1.1 μg/mL), while CORAGEN remained unaffected (LC_90_ = 7.2 μg/mL). Overall, starvation tended to enhance the potency of most insecticides against RPW larvae (**Table 9**).

**Table 9:** Variation in LC_50_ and LC_90_ values of ten insecticides against RPW larvae under fed and starved laboratory conditions.

Insecticides	LC_50_			LC_90_		
	Without food	With food	Difference	Without food	With wood	Difference
CORAGEN	3.5	4.8	+1.3	7.2	7.2	0
SIVANTO	2.1	2.0	-0.1	4.5	4.5	0
Voliam Flexi	1.8	2.8	+1.0	3.9	5.0	+1.1
FEDOTHRIN	6.5	9.6	+3.1	12.8	13.2	+0.4
FIPROL	1.2	1.2	0	2.8	3.0	+0.2
MEDPRID	2.8	1.8	-1.0	5.6	3.6	-2.0
Cyprone	2.4	3.5	+1.1	4.9	6.0	+1.1
INDOCARB	4.2	11.5	+7.3	8.5	15.0	+6.5
LAMDOC	3.0	4.0	+1.0	6.2	6.5	+0.3
DECIBAN	3.8	5.0	+1.2	7.9	8.0	+0.1

In adult RPW, the influence of feeding was generally less pronounced than in larvae, though significant differences were still observed for certain compounds. CORAGEN, SIVANTO, and FEDOTHRIN exhibited moderately higher LC_50_ and LC_90_ values under fed conditions, with differences ranging from 0.36 to 3.55 μg/mL, indicating reduced efficacy in fed adults. Interestingly, Cyprone and INDOCARB displayed a reversed pattern, with LC_50_ and LC_90_ slightly lower under starvation, suggesting a complex interaction between feeding status and adult metabolism. Overall, adult susceptibility was less dramatically influenced by starvation compared to larvae, though some insecticides, notably CORAGEN and SIVANTO, still showed clear feeding-related differences (**Table 10**).

**Table 10:** Variation in LC_50_ and LC_90_ values of ten insecticides against RPW adults under fed and starved laboratory conditions.

Insecticides	LC_50_ without food	LC_90_ with food	Difference (Without – With)	LC_50_ without food	LC_90_ fith food	Difference (Without – With)
CORAGEN	4.86	4.18	0.68	13.74	10.36	3.38
SIVANTO	4.62	3.25	1.37	12.05	8.50	3.55
Voliam Flexi	3.15	2.89	0.26	6.82	6.23	0.59
FEDOTHRIN	6.03	5.67	0.36	14.27	13.24	1.03
FIPROL	2.21	2.12	0.09	5.84	5.54	0.30
MEDPRID	2.89	2.54	0.35	7.23	6.87	0.36
Cyprone	3.65	4.56	-0.91	8.94	11.23	-2.29
INDOCARB	5.31	6.31	-1.00	13.02	14.52	-1.50
LAMDOC	4.12	4.92	-0.80	9.76	12.05	-2.29
DECIBAN	3.88	3.74	0.14	9.35	9.18	0.17

Direct comparison between larvae and adults highlights that larval susceptibility is generally more sensitive to feeding status than adult susceptibility. In larvae, starvation increased susceptibility for most insecticides, often with substantial differences in LC_50_ and LC_90_ values, whereas adults showed more modest and variable responses. Notably, INDOCARB and FEDOTHRIN were highly affected by feeding in both stages, but the magnitude of effect was considerably higher in larvae. These results suggest that larval physiology and feeding behavior strongly modulate insecticide toxicity, whereas adult RPW may possess compensatory mechanisms that reduce the impact of starvation on susceptibility.

## 4 Discussion

Some studies highlight how feeding status strongly shapes the biochemical and histological responses of RPW larvae to insecticides (24, 27). Actively feeding larvae absorbed higher doses through ingested tissue, resulting in strong inhibition of acetylcholinesterase and disruption of antioxidant enzymes, with spinosad causing the most severe effects (35). Chlorpyrifos triggered oxidative stress and catalase activity, while methomyl and spinosad suppressed key antioxidant defenses, making detoxification more difficult. Importantly, starvation reduces metabolic reserves, thereby intensifying oxidative damage and increasing mortality risk (25, 26). Histological lesions, such as vacuolar degeneration, necrosis, and basement membrane destruction, further impair digestion and nutrient uptake, mimicking starvation-like conditions (36, 37). Overall, these results show that feeding accelerates insecticide uptake, while starvation magnifies vulnerability, underscoring the need to integrate nutritional status into pest control strategies.

The present findings demonstrate clear differences between larvae and adults of R. ferrugineus in their response to starvation. While larvae showed greater vulnerability, significant reductions in total proteins (31–50%) and carbohydrates (35–49%) after 48–120 hours were evident, adult responses were comparatively moderate (5–22% for proteins and 41–46% for carbohydrates). Importantly, starved larvae failed to recover activity after refeeding, suggesting irreversible physiological stress, whereas adults were able to regain weight and biochemical balance upon refeeding (18).

These results align with earlier studies on insect energy metabolism, where glycogen and trehalose reserves were rapidly depleted under starvation to fuel essential metabolic functions. To further substantiate our mechanistic claims, future studies should incorporate measurements of detoxification markers such as P450 activity and basic energy reserves like glycogen, lipids, and proteins for fed versus starved insects. The observed rise in triglycerides and glutamic-pyruvic transaminase (GPT) activity under starvation reflects metabolic shifts toward lipid mobilization and protein catabolism, which is a survival strategy reported in other insects such as *Tribolium castaneum* and *Harmonia axyridis* (38, 39). The greater starvation resistance of adults, compared with larvae, may explain the persistence of R. ferrugineus populations under unfavorable conditions, such as food scarcity during off-seasons or following partial sanitation practices (25, 26). These findings suggest that larvae represent the weakest stage under starvation stress, a vulnerability that could be targeted in pest control strategies. For instance, management tactics that prolong starvation by disrupting larval access to palm tissues or integrating starvation stress into biological agents that could enhance more control efficacy (25, 26). Overall, starvation significantly impairs the metabolic stability of R. ferrugineus, particularly in larvae, highlighting the importance of nutritional ecology in shaping pest survival. Exploiting starvation-induced physiological weakness may provide a complementary tool to chemical and biological methods for reducing palm losses (25, 26, 40).

This study demonstrates that insecticide efficacy against R. ferrugineus varies significantly between larvae and adults under starvation, highlighting the role of feeding status in toxicity outcomes (25, 26). The findings emphasize that formulation, concentration, and nutritional condition critically shape mortality responses, offering valuable guidance for refining chemical control within IPM programs (40). Among all tested insecticides, Voliam Flexi demonstrated the highest efficacy, achieving 100% mortality across all concentrations and feeding conditions. Its active ingredient, chlorantraniliprole, targets ryanodine receptors, disrupting calcium balance and causing rapid paralysis and death in larvae (41). In contrast, Coragen 20SC, Cyprone 250EC, and Indocarb 150SC required double doses to reach full mortality, indicating dose-dependent effects consistent with prior findings on indoxacarb-based insecticides (34). Fedothrin 10% EC and Deciban 25EC showed moderate results (~80%), suggesting they are better suited for rotation or combination within IPM frameworks (41, 42). Fiprol 50SC, Medprid 35%SC, and Lamdoc 50EC improved with higher doses but failed to achieve total kill, reflecting lower toxicity or slower action (43). Statistical analyses confirmed significant variation among treatments, highlighting Voliam Flexi, Coragen, and Sivanto as top performers.

Feeding status also influenced toxicity—starvation enhanced efficacy for Cyprone and Indocarb, while feeding reduced it for Sivanto and Coragen. To confirm these observations, future research should assess the relationship between starvation and detoxification capacities, possibly measuring P450 enzyme activity and energy reserves. Overall, targeting starved larvae with potent formulations like Voliam Flexi and optimizing dosage and timing can greatly enhance RPW control while supporting sustainable IPM programs (27, 36).

In *R. ferrugineus*, gut microbial communities play a vital role in digestion, immunity, and detoxification, helping the insect degrade toxic compounds and develop tolerance to insecticides (44). The presence of food likely enhances microbial activity, delaying mortality by reducing insecticide uptake or promoting its breakdown. Larvae showed greater resistance than adults, possibly due to their richer and more diverse gut microbiota, which supports high metabolic demand and detoxification during development (44). The significantly higher mortality in both larvae and adults of R. ferrugineus exposed to insecticides without food suggests that the gut microbiota plays a crucial role in regulating insecticide susceptibility. The presence of food likely facilitates microbially mediated detoxification processes and thereby reduces the toxicity of insecticides (44). This phenomenon is increasingly recognized in insect physiology, where symbiotic microorganisms in the gut act as a biochemical shield, metabolizing or neutralizing xenobiotics before they reach their target sites (39, 40).

Comparative analysis of dose-response dynamics in R. ferrugineus adults and larvae under fed and starved conditions revealed that starvation significantly increased the toxicity of insecticides, especially in larvae (39, 40, 44, 45). Starved larvae showed significantly lower LC_50_ and LC_90_ values for several insecticides (e.g., CORAGEN, FEDOTHRIN, and INDOCARB), indicating a reduced detoxification capacity, probably due to a disturbed gut microbiota (40). In contrast, RPW adults showed relatively stable responses regardless of feeding status, suggesting less dependence on microbially mediated detoxification and slower metabolism (47, 48). Additionally, probit regression analysis revealed steeper slopes in fed larvae, suggesting a more consistent toxicological response compared to the variable responses observed during starvation (49–52).

The susceptibility of *R. ferrugineus* to insecticides differs markedly between larval and adult stages, largely due to their distinct feeding behaviors and habitats (7, 28). Larvae, which feed continuously within date palm tissues, are highly influenced by the presence of plant material during insecticide exposure (24, 28, 45, 46). This internal feeding can reduce the effectiveness of chemical treatments by limiting direct contact or absorption (28). Research indicates that the interaction between insecticide type and food availability plays a key role in larval mortality (28, 53, 54). For example, studies on chlorpyrifos, methomyl (28), and spinosad demonstrated that sub-lethal exposures, such as larvae feeding on insecticide-treated sugarcane stems, induce significant biochemical and histological changes (24, 55). Reported LC50 values for chlorpyrifos, methomyl, and spinosad were 109.73, 589.55, and 112.09 μg a.i./ml, respectively, highlighting differences in toxicity through ingestion (28, 56, 57). While continuous feeding provides larvae with prolonged exposure to insecticides, it also offers a protective barrier against direct contact, emphasizing the need for systemic or ingestion-based delivery for effective control. In contrast, adult RPWs live externally and feed intermittently, so their feeding status has little direct influence on insecticide-induced mortality (28, 58). Although the interaction between insecticide type and feeding condition remains relevant, its impact is notably weaker in adults than in larvae (28, 58, 59), reflecting adults’ lower reliance on immediate food intake for susceptibility.

The effectiveness of insecticides against R. ferrugineus is influenced by a complex interplay of factors, including the insect’s developmental stage, feeding behavior, symbiotic microbial communities, physiological metabolism, and genetic variability within populations (1, 17, 55–57, 60, 61). A comprehensive understanding of these determinants is essential for optimizing insecticide application strategies and mitigating the risk of resistance development. The gut microbiota critically influences insecticide susceptibility in insects (56, 57). Symbionts can directly degrade insecticidal compounds or enhance the host’s detoxification pathways, sometimes leading to field control failures (8, 61, 62). In invasive pests like R. ferrugineus, gut microbes also affect nutrient assimilation and adaptation to new food sources (56, 63, 64). Microbial communities can therefore either amplify or reduce insecticide toxicity, shaping overall treatment effectiveness. Besides, insecticide sensitivity and resistance are also strongly influenced by detoxification enzymes (59, 65). P450 monooxygenases, GSTs, and esterases metabolize various insecticides, with gene expression levels (e.g., CYP6AB14, CYP9A98) varying under different treatments, reflecting their key role in metabolic defense (59, 60, 66).

Genetic diversity within insect populations plays a fundamental role in shaping insecticide efficacy and the evolutionary trajectories of resistance (1, 52, 61). In R. ferrugineus, substantial variation has been documented; for instance, 53 haplotypes were identified in the Qassim region alone (1, 52), suggesting that population-level genetic variation can modulate detoxification enzyme profiles and influence responses to different insecticide types and doses (52, 62). Population genetic frameworks are essential to explain the variation in insecticide efficacy and how environmental factors, feeding status, and fitness costs collectively drive resistance outcomes (58, 59). Resistance evolution in RPW is largely accelerated by indiscriminate insecticide use and persistent selection pressure (55, 61, 66–68). Multiple mechanisms may underline this process, including behavioral avoidance, reduced cuticular penetration, enhanced metabolic detoxification, and target-site modifications. This highlights the importance of considering genetic structure and selection dynamics when designing insecticide-based management strategies for RPW (69, 70).

In contrast, when red palm weevils are starved or not feeding, they generally show lower susceptibility to insecticides. This may occur due to reduced feeding, which lowers the amount of toxin they ingest and slows their metabolic activity (24, 26). Starvation tends to down-regulate key detoxification enzyme systems and reduces contact with treated surfaces, resulting in lower overall mortality rates (26, 70, 71). These feeding-dependent differences emphasize the importance of timing insecticide applications during periods of active feeding to achieve maximum impact and integrating behavioral and physiological knowledge into RPW control programs. However, despite this clear relationship between feeding status and insecticide sensitivity, more research is still required to understand precisely how starvation, partial feeding, or re-feeding affects detoxification pathways, enzyme regulation, and molecular responses at different life stages. Such insights could help improve field-level predictions of insecticide performance and support better timing strategies for targeted interventions.

Insects, such as RPW, rely on complex detoxification pathways to metabolize both insecticides and plant secondary chemicals (63, 72). These pathways mainly involve three major enzyme groups: P450s, GSTs, and carboxylesterases (64–66). P450s are particularly critical during Phase I detoxification, where they drive oxidation, reduction, and hydrolysis reactions (64–66). Changes in the activity of these enzymes are strongly linked with either susceptibility or resistance (67, 68). For example, exposure to bifenthrin or pirimiphos-methyl alters esterase profiles in Sitophilus oryzae and S. zeamais (69, 72), while shifts in esterase, GST, and P450 activity have been associated with variable insecticide responses in stink bugs (63).

The physiological condition of RPW itself is also an important factor shaping toxicological outcomes (63). Starvation is a major stressor that rapidly depletes energy reserves and alters metabolism (70). In pea aphids, for example, starvation markedly reduces glycogen and triglyceride levels (66). When energy is limited, the insect may not be able to sustain detoxification, which is an ATP- and NADPH-dependent process (68). This suggests that starvation could weaken the insect’s biochemical defense machinery against insecticides (67). Behavioral resistance is an additional factor that can influence the performance of insecticides, as some insects can simply avoid treated surfaces or change their movement patterns (70, 71). Yet this is fundamentally different from the physiological shifts caused by starvation. Previous work in insect physiology and toxicology provides several clues that support this idea. Studies have shown, for instance, that some plant phenylpropanoids have larvicidal effects on RPW and can slow larval development by altering the expression of detoxification-related genes (72, 73). These findings imply that the detoxification system in RPW is plastic and responds to outside pressures, including changes in diet and energy availability. Lack of food can rewire metabolism and modify olfactory behavior, altering the insect’s attraction to hosts or pheromone cues, which in turn may indirectly change their likelihood of encountering an insecticide (74, 75).

This study demonstrates that the nutritional status of RPW strongly affects its response to insecticides: when insects are starved and their energy reserves decline, detoxification enzymes become less active, toxicants accumulate faster, and mortality increases even at lower doses. In contrast, feeding supports metabolic activity and temporarily strengthens detox capacity. These findings indicate that chemical treatments could be more effective if applied during natural starvation periods, such as after the removal of soft tissues in infested palms or before food is offered in trap-and-kill systems. This approach allows lower doses to achieve higher impact, reduces chemical load, slows resistance development, and improves IPM sustainability.

Finally, by establishing this mechanistic link between starvation and toxicity, this work introduces a new direction in RPW management and provides a practical foundation for precision timing of insecticide applications based on the weevil’s physiological condition, rather than relying solely on descriptive toxicity metrics.

## 5 Conclusions

The susceptibility of *R. ferrugineus* to insecticides is strongly influenced by developmental stage and feeding status, with starved larvae being the most vulnerable. In this study, Voliam Flexi demonstrated the highest efficacy, achieving near-complete mortality even at low doses in both starved and fed larvae and adults. CORAGEN was effective in unfed (starved) larvae and fed adults, while SIVANTO showed higher efficacy in unfed adults. Fed larvae exhibited enhanced detoxification and reduced insecticide efficacy. Adults showed moderate responses, with unfed adults slightly increasing susceptibility.

Unfed status (starvation) deplete energy and antioxidant reserves, weakening detoxification systems and disrupting gut microbial symbiosis, which can be exploited in IPM through larval access restriction, removal of infested tissue, or introduction of biological agents. Genetic diversity, including mutations in Qassim populations, further influences susceptibility, highlighting the need for population-specific strategies. The study recommends prioritizing feeding-independent insecticides, timing applications to coincide with starvation periods, carefully using feeding-sensitive compounds with complementary measures, and integrating nutritional and microbial stress interventions. Incorporating genetic and microbial profiling can further optimize IPM and mitigate resistance. Targeting the physiological and ecological vulnerabilities of RPW, particularly starved larvae, provides a precision-based, sustainable approach to enhance control efficiency, reduce insecticide use, and minimize economic losses.

In conclusion, the most effective insecticides for each life stage and feeding condition are clearly delineated. Voliam Flexi achieved the highest mortality in fed larvae, while both Voliam Flexi and CORAGEN were highly effective against starved larvae. For adults, Voliam Flexi and CORAGEN performed best in fed individuals, whereas Voliam Flexi and SIVANTO exhibited superior efficacy in starved adults. These results highlight the critical role of feeding status and developmental stage in determining insecticide susceptibility and reinforce the strategic value of feeding-independent compounds in the design of robust and targeted IPM programs.

## Data Availability

The raw data supporting the conclusions of this article will be made available by the authors, without undue reservation.
